# Clinical implications of the novel cytokine IL-38 expressed in lung adenocarcinoma: Possible association with PD-L1 expression

**DOI:** 10.1371/journal.pone.0181598

**Published:** 2017-07-20

**Authors:** Kazuki Takada, Tatsuro Okamoto, Masaki Tominaga, Koji Teraishi, Takaki Akamine, Shinkichi Takamori, Masakazu Katsura, Gouji Toyokawa, Fumihiro Shoji, Masaki Okamoto, Yoshinao Oda, Tomoaki Hoshino, Yoshihiko Maehara

**Affiliations:** 1 Department of Surgery and Science, Graduate School of Medical Sciences, Kyushu University, Fukuoka, Japan; 2 Department of Anatomic Pathology, Graduate School of Medical Sciences, Kyushu University, Fukuoka, Japan; 3 Division of Respirology, Neurology, and Rheumatology, Department of Internal Medicine, Kurume University School of Medicine, Kurume, Fukuoka, Japan; National Cancer Center, JAPAN

## Abstract

Interleukin (IL)-38, a novel member of the IL-1 cytokine family, is homologous to IL-1 receptor antagonist (IL-1Ra) and IL-36Ra, and has been reported to act as an antagonist. IL-38 expression is found in tonsil, placenta, and spleen, and recent studies suggest an association between IL-38 and autoimmune diseases. However, whether IL-38 plays a role in carcinogenesis or cancer growth is unclear. In the present study, we identified increases in IL-38 expression by immunohistochemistry in multiple types of cancer cells. In the examination of 417 surgically resected primary lung adenocarcinomas, Fisher’s exact tests showed significant associations between high IL-38 expression and high tumor grades, an advanced T status, advanced N status, advanced stage, and the presence of pleural and vessel invasions. Survival analyses by the Kaplan-Meier method showed that patients with high expression of IL-38 had significantly shorter disease-free survival and shorter overall survival after surgery than patients with low expression of IL-38 (log-rank test: *P* = 0.0021 and *P* = 0.0035, respectively). Moreover, programmed cell death-ligand 1 (PD-L1)-positive cases showed higher expression of IL-38 than PD-L1-negative cases (Wilcoxon rank-sum test: *P* < 0.0001). In conclusion, IL-38 was expressed in tumor cells of various cancers, and IL-38 expression was associated with poor survival of lung adenocarcinoma patients. IL-38 may affect host immunity or the tumor microenvironment, and contribute to the progression of lung adenocarcinoma.

## Introduction

Immunotherapy targeting programmed cell death-1 (PD-1) and programmed cell death-ligand 1 (PD-L1) has recently been shown to improve prognoses of multiple cancer types [1, 2]. Notably, in non-small cell lung cancer (NSCLC) patients, PD-1 inhibitors, such as nivolumab in CheckMate and pembrolizumab in KEYNOTE studies, and PD-L1 inhibitors, including atezolizumab in POPLAR and OAK studies, have exhibited a survival benefit compared with conventional standard therapy [3–8]. PD-1 inhibitors have become standard treatment for NSCLC patients after failure of first-line chemotherapy. PD-L1 expression on tumor cells, which might serve as a predictive marker for PD-1/PD-L1 inhibitors, is regulated by endogenous antitumor immunity and reflects an immune-active microenvironment [9, 10]. Expression of PD-L1 on tumor cells might be mainly regulated by the microenvironment around tumor cells, such as various cytokines from tumor-infiltrating immune cells and immunosuppressive factors. Therefore, many researchers are interested in the tumor microenvironment, and many studies concerning the tumor microenvironment have been reported.

Interleukin (IL)-38 is a novel cytokine of the IL-1 family, and its expression has been reported in skin, tonsil, thymus, spleen, fetal liver, placenta, and salivary glands [11–15]. IL-38 shares 41% homology with IL-1 receptor antagonist (IL-1Ra) and 43% homology with IL-36Ra, and may act as an IL-1 family antagonist [11–14]. It may participate in a network of IL-1 family members to regulate adaptive and innate immune responses. IL-38 plays a role in the pathogenesis of inflammatory diseases, exerting a protective effect against some autoimmune diseases or nonneoplastic diseases [16–24]. However, whether IL-38 plays a role in carcinogenesis or cancer growth is unclear. IL-38 may affect host immunity or the tumor microenvironment because it is a negative regulator functionally related to receptor antagonists and involved in human inflammation and autoimmunity.

In this translational study, we identified increases in IL-38 expression of tumor cells in multiple cancer types, including NSCLC, by immunohistochemistry. Therefore, we examined IL-38 expression in 417 surgically resected primary lung adenocarcinomas by immunohistochemistry, and investigated the associations of IL-38 expression with clinicopathological features and patient outcomes. Moreover, we investigated the relationship between PD-L1 and IL-38 expression. This study is the first report concerning the relevance between IL-38 expression and the prognosis of malignant tumors.

## Materials and methods

### Patients and samples

We retrospectively examined patients who underwent surgical resection of their primary lung adenocarcinoma between January 2003 and December 2012 at the Department of Surgery and Science, Graduate School of Medical Sciences, Kyushu University. Thirteen patients who had received neoadjuvant therapy and five patients with stage IV disease were excluded. Finally, 417 paraffin-embedded specimens were available and retrieved from the registry of the Department of Anatomic Pathology, Graduate School of Medical Sciences, Kyushu University. Clinicopathological features, including age at surgery, sex, smoking history, tumor differentiation, pathological tumor-node-metastasis (TNM) stage (seventh edition of the lung cancer staging system), pleural or lymphovascular invasion, histological subtype (World Health Organization Classification 2015), surgical procedure, and *epidermal growth factor receptor* (*EGFR*) mutation status, were examined. *EGFR* status had been determined in tumor tissues using the peptide nucleic acid-locked nucleic acid (PNA-LNA) polymerase chain reaction clamp method (Mitsubishi Chemical Medience, Tokyo, Japan) in 235 specimens [25]. Briefly, systemic dissection of hilar and mediastinal lymph nodes was performed at the same time as pulmonary lobectomy. Selected lymph node sampling was performed during sublobar resection. Perioperative therapy, which was selected by the physician, was performed within the clinical practice guidelines for lung cancer in Japan. An *EGFR*-tyrosine kinase inhibitor was used after recurrence in patients with tumors harboring *EGFR*-sensitive mutations. After surgery, routine checkups, including a physical examination, blood tests (including serum tumor markers), and chest x-ray, were performed at 3 month intervals for the first 3 years and at 6 month intervals thereafter. Computed tomography was performed twice a year for the first 3 years and then at least annually thereafter. Adjuvant chemotherapy was administered to some patients when the treatment was required. The eligibility criteria for patients receiving adjuvant chemotherapy were as follows: (i) p-stage IB to IIIA disease, (ii) less than 76 years of age, (iii) performance status of 0 or 1, and (iv) provided written informed consent. The regimen for p-stage IB disease was uracil-tegafur, and that for p-stage IIA to IIIA disease was a platinum-based combined regimen in principle. Clinical information and follow-up data were obtained from medical records. This study was approved by our institutional review board (Kyushu University, IRB No. 27–131).

### Immunohistochemical analysis

To determine the expression of IL-38 and PD-L1 in human tumors tissues immunohistochemically, we used formalin-fixed and paraffin-embedded (FFPE) tumor tissue sections. The immunohistochemical analysis was conducted using a non-commercial antibody against IL-38 (mouse monoclonal, clone H127C, 0.5 μg/ml, kindly provided by T. Hoshino, Kurume University, Fukuoka, Japan) [26] and a commercial antibody against PD-L1 (rabbit monoclonal, clone SP142, 1:100 dilution, Spring Bioscience, Ventana, Tucson, AZ, USA). Immunohistochemical staining of PD-L1 was performed as described previously [27–29]. For immunohistochemical staining of IL-38, sections were prepared (4 μm thick), dewaxed with xylene, and rehydrated through a graded series of ethanol solutions. After inhibition of endogenous peroxidase activity by treatment with 3% H_2_O_2_ in methanol for 30 min, the sections were treated with pH 6.0 citrate buffer in a decloaking chamber at 110°C for 15 min. Protein blocking was performed with Dako serum-free protein block for 20 min, and then the sections were incubated with monoclonal antibodies at 4°C overnight. Immune complexes were detected with a DAKO EnVision Detection System (Dako). The sections were finally reacted in 3,3′-diaminobenzidine, counterstained with hematoxylin, and mounted. Sections from human tonsil, placenta, and spleen tissues were used as positive controls for IL-38, and the diluted solution containing both the antibody against IL-38 and IL-38 recombinant protein (60 μg/ml) was used in place of that containing only the antibody against IL-38 for the negative staining control.

Immunohistochemical evaluation of PD-L1 was conducted as described previously [29]. Cases with less than 5% tumor membrane staining were considered as negative in this study. In terms of IL-38, cytoplasm or membrane immunostaining were quantified in all tumor cells of all cases, and immunohistochemical evaluation was performed as follows. Briefly, the intensity was scored from 1 to 3 (1, weak staining; 2, moderate staining; 3, strong staining), and cases with score 2 or 3 were judged as high expression because IL-38 protein was expressed in all tumor cells with the same intensity in a case and the proportion was 100% in all cases. All immunohistochemical results in this study were judged by three investigators who were blinded to the clinical status of the patients. A consensus judgment was adopted as the final result.

### Statistical analysis

Associations between IL-38 expression and patient characteristics were analyzed using Fisher’s exact test. Univariate and multivariate analyses of the relationship between IL-38 expression and other patient characteristics were performed by logistic regression analysis with the backward elimination method. Disease-free survival (DFS) was defined as the period between surgery and the date of recurrence, and overall survival (OS) was defined as the period between surgery and the date of the last follow-up or death. These rates were estimated using the Kaplan-Meier method with the log-rank test. Cox proportional hazards regression analysis was performed to estimate the hazard ratios for positive risk factors with the backward elimination method. The association between PD-L1 and IL-38 expression was examined with the Wilcoxon rank-sum test. All statistical analyses were performed using JMP Statistical Discovery Software (version 11.0; SAS Institute, Cary, NC). Results were considered as statistically significant at *P* < 0.05.

## Results

### Expression of IL-38 in tumor cells of multiple cancer types

**Fig 1:**
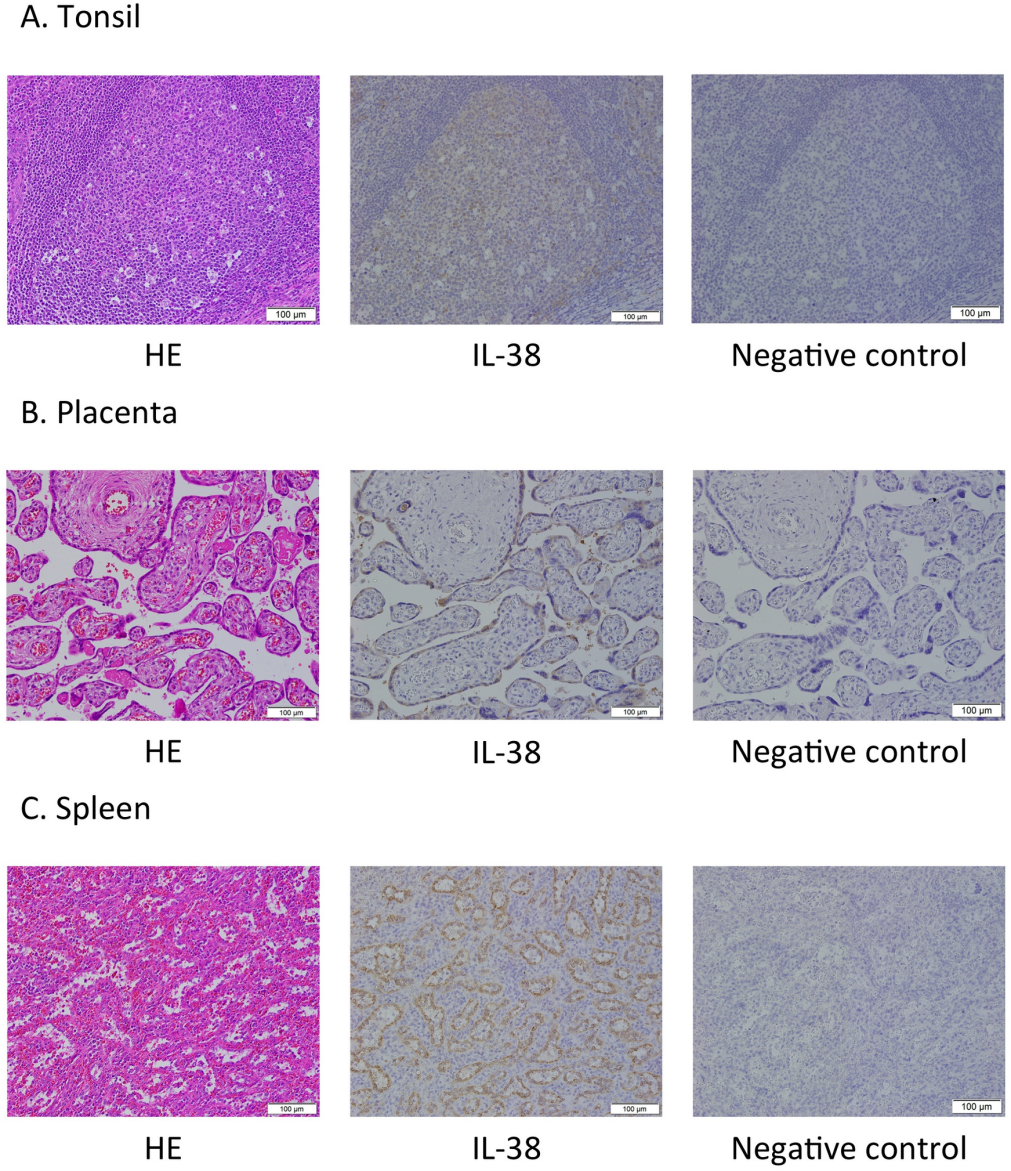
Representative images of (left panel) HE staining, (middle panel) IL-38 staining, and (right panel) negative controls. (A) Human tonsil tissue. (B) Human placenta tissue. (C) Human spleen tissue. HE: Hematoxylin-eosin, IL-38: interleukin-38. Scale bar: 100 μm.

First, we conducted a pilot study using FFPE tumor tissue sections (lung adenocarcinoma, lung squamous cell carcinoma, lung small cell carcinoma, esophageal cancer, gastric cancer, colon cancer, hepatocellular carcinoma, and breast cancer) with positive and negative controls. We examined IL-38 expression in 10 cases each (total 80 cases) by immunohistochemistry, and identified increases in IL-38 expression of tumor cells in multiple cancer types (**Fig 1**, **S1 Fig**).

### Association between IL-38 expression and clinicopathological characteristics in patients with primary lung adenocarcinoma

Based on the above results, we speculated that IL-38 may affect host immunity or the tumor microenvironment. We focused primary on lung adenocarcinomas and examined IL-38 expression in 417 surgically resected primary lung adenocarcinomas by immunohistochemistry.

A total of 417 patients with primary lung adenocarcinoma who underwent surgical resection were included in the present study (**Table 1**). Two hundred and five (49.2%) patients were male, and 218 (52.3%) had never smoked. The median age of all patients was 69 years (range, 29–85 years). *EGFR* status was available for 235 patients of which 123 (52.3%) had wild-type *EGFR* and 112 (47.7%) had mutant *EGFR*.

**Table 1:** Clinicopathological characteristics of patients with primary lung adenocarcinoma.

Factors		Value or no. of patients
Age (years)	Median	69
	Range	29–85
Sex	Male	205
	Female	212
Smoking status	Never smoked	218
	Smoker	199
Grade	G1	202
	G2	158
	G3	57
T	T1	247
	T2	138
	T3	22
	T4	10
N	N0	337
	N1–3	80
Stage	I	305
	II	63
	III	49
	IV	0
pl	No	323
	Yes	94
ly	No	357
	Yes	60
v	No	300
	Yes	117
Histological subtype	AAH/AIS/MIA	42
	Lepidic predominant	26
	Papillary predominant	307
	Acinar predominant	7
	Micropapillary predominant	1
	Solid predominant	26
	Variants	8
Surgical procedure	Lobectomy	310
	Bilobectomy	4
	Pneumonectomy	3
	Sublobar resection	100
EGFR*	Wild-type	123
	Mutant	112

*cases for which data were available.

pl: pleural invasion, ly: lymphatic invasion, v: vascular invasion, *EGFR*: epidermal growth factor receptor gene, AAH: atypical adenomatous hyperplasia, AIS: adenocarcinoma in situ, MIA: minimally invasive adenocarcinoma.

**Fig 2:**
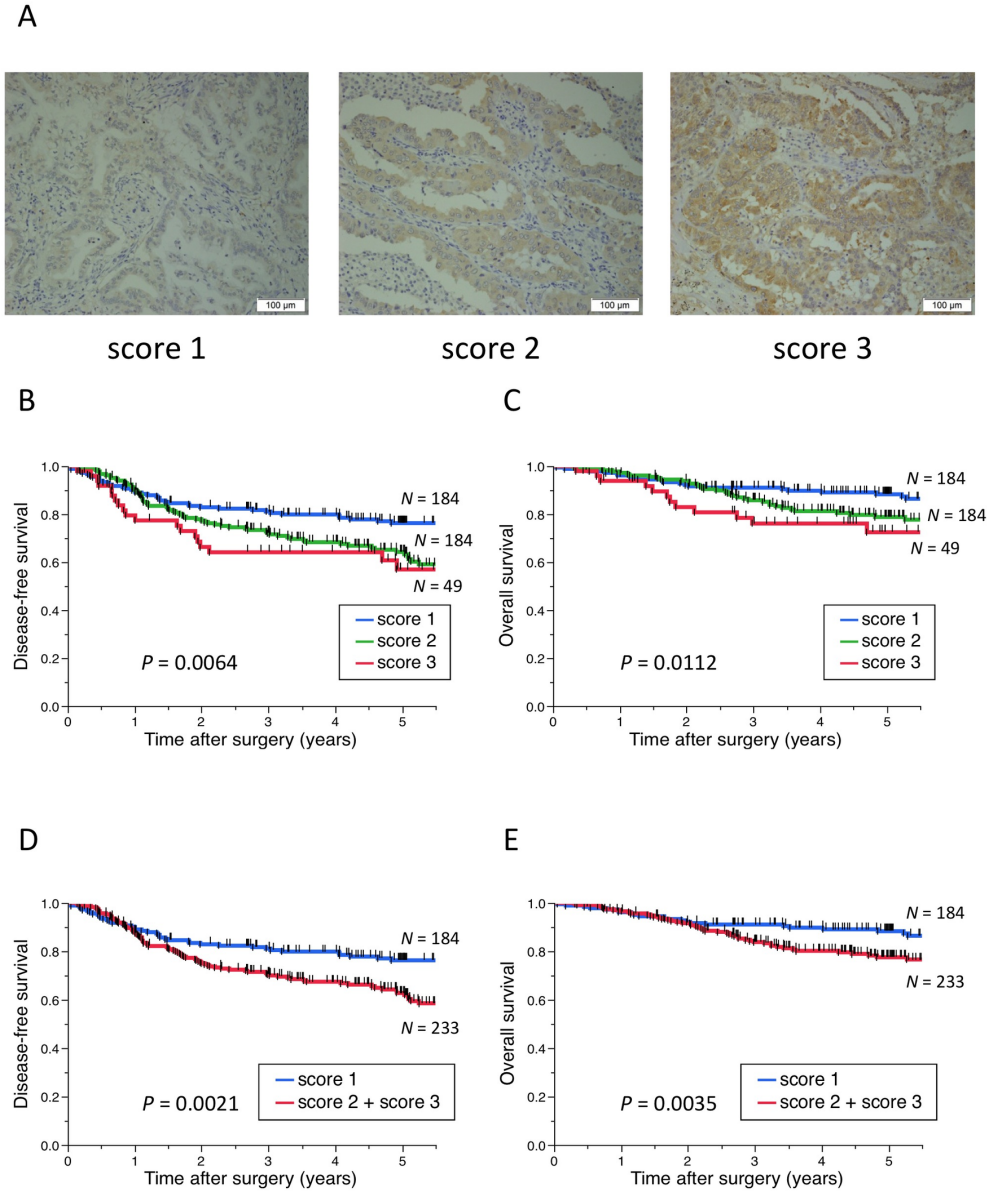
Representative images of IL-38 expression and Kaplan-Meier curves according to IL-38 expression in patients with primary lung adenocarcinoma. (A) Representative images of IL-38 expression showing (left panel) score 1, (middle panel) score 2, and (right panel) score 3 in primary lung adenocarcinoma. (B–E) Kaplan-Meier curves showing the (B, D) disease-free and (C, E) overall survival of primary lung adenocarcinoma patients according to IL-38 expression. IL-38: interleukin-38. Scale bar: 100 μm.

Immunohistochemical staining for IL-38 was detected in the cytoplasm or membrane of carcinoma cells (**Fig 2A**). **Table 2**shows the associations between IL-38 expression and patient clinicopathological characteristics. The samples from 184 (44.1%), 184 (44.1%), and 49 (11.8%) patients were judged to be score 1, 2, and 3, respectively, and the samples from 233 (55.9%) patients were high expression. Fisher’s exact tests showed that high expression of IL-38 was significantly associated with high tumor grades, an advanced T status, advanced N status, advanced stage, and the presence of pleural and vessel invasions. Multivariate analysis revealed that a high pathological grade and the presence of vessel invasion were independent predictors for high expression of IL-38 (**Table 3**).

**Table 2:** Association between IL-38 expression and clinicopathological factors in patients with primary lung adenocarcinoma.

Factors		*N*	IL-38, *N* (%)		*P*-value
			Low	High	
Age (years)	<70	222	99 (53.8)	123 (52.8)	0.8440
	≥70	195	85 (46.2)	110 (47.2)	
Sex	Male	205	85 (46.2)	120 (51.5)	0.3240
	Female	212	99 (53.8)	113 (48.5)	
Smoking status	Never smoked	218	106 (57.6)	112 (48.1)	0.0607
	Smoker	199	78 (42.4)	121 (51.9)	
Grade	G1	202	108 (58.7)	94 (40.3)	0.0003
	≥G2	215	76 (41.3)	139 (59.7)	
T	T1	247	119 (64.7)	128 (54.9)	0.0456
	≥T2	170	65 (35.3)	105 (45.1)	
N	N0	337	161 (87.5)	176 (75.5)	0.0025
	≥N1	80	23 (12.5)	57 (24.5)	
Stage	Ⅰ	305	149 (81.0)	156 (67.0)	0.0018
	≥II (II/III)	112 (63/49)	35 (19.0)	77 (33.0)	
pl	No	323	153 (83.2)	170 (73.0)	0.0135
	Yes	94	31 (16.8)	63 (27.0)	
ly	No	357	162 (88.0)	195 (83.7)	0.2609
	Yes	60	22 (12.0)	38 (16.3)	
v	No	300	149 (81.0)	151 (64.8)	0.0003
	Yes	117	35 (19.0)	82 (35.2)	
Histological subtype	Micropapillary/solid	27	8 (4.3)	19 (8.2)	0.1599
	Others	390	176 (95.7)	214 (91.8)	
Surgical procedure	≥Lobectomy	317	138 (75.0)	179 (76.8)	0.7292
	Sublobar resection	100	46 (25.0)	54 (23.2)	
EGFR*	Wild-type	123	45 (50.6)	78 (53.4)	0.6882
	Mutant	112	44 (49.4)	68 (46.6)	

*cases for which data were available.

*P*-values for Fisher’s exact test.

IL-38: interleukin-38, pl: pleural invasion, ly: lymphatic invasion, v: vascular invasion, *EGFR*: epidermal growth factor receptor gene.

**Table 3:** Univariate and multivariate analyses of the relationship between IL-38 expression and other patient characteristics.

Factors		Univariate analysis		Multivariate analysis	
		OR (95% CI)	*P*-value	OR (95% CI)	*P*-value
Age (years)	≥70/<70	1.04 (0.71–1.54)	0.8366		
Sex	Male/female	1.24 (0.84–1.82)	0.2817		
Smoking status	Smoker/never smoked	1.47 (0.99–2.17)	0.0525		
Grade	≥G2/G1	2.10 (1.42–3.12)	0.0002	1.65 (1.05–2.59)	0.0300
Stage	≥II/I	2.10 (1.34–3.35)	0.0012		
pl	Yes/no	1.83 (1.14–2.99)	0.0125		
ly	Yes/no	1.43 (0.82–2.56)	0.2056		
v	Yes/no	2.31 (1.48–3.68)	0.0002	1.75 (1.04–2.96)	0.0351
Histological subtype	Micropapillary or solid/others	1.95 (0.86–4.83)	0.1102		
Surgical procedure	≥Lobectomy/sublobar resection	1.10 (0.70–1.73)	0.6652		
EGFR*	Wild-type/mutant	1.12 (0.66–1.90)	0.6700		

*cases for which data were available.

*P*-values for logistic regression analysis.

IL-38: interleukin-38, pl: pleural invasion, ly: lymphatic invasion, v: vascular invasion, *EGFR*: epidermal growth factor receptor gene, OR: odds ratio, CI: confidence interval.

### Univariate and multivariate survival analyses of surgically resected primary lung adenocarcinoma according to IL-38 expression

We assessed the associations between IL-38 expression and postoperative survival of patients. Survival analyses by the Kaplan-Meier method showed that patients with higher expression of IL-38 had significantly shorter DFS and shorter OS after surgery (log-rank test: *P* = 0.0064 and *P* = 0.0112, respectively, **Fig 2B and 2C**). Furthermore, patients with high expression of IL-38 had significantly shorter DFS and shorter OS after surgery than patients with low expression of IL-38 (log-rank test: *P* = 0.0021 and *P* = 0.0035, respectively, **Fig 2D and 2E**).

In the multivariate analysis, age, stage, and lymphatic invasion remained as predictors for both DFS and OS, but high expression of IL-38 did not remain as a predictor for DFS and OS (**Table 4**).

**Table 4:** Univariate and multivariate analyses of DFS and OS in patients with primary lung adenocarcinoma.

Factors		DFS				OS			
		Univariate analysis		Multivariate analysis		Univariate analysis		Multivariate analysis	
		HR (95%CI)	*P* value	HR (95%CI)	*P* value	HR (95%CI)	*P* value	HR (95%CI)	*P* value
Age (years)	≥70/<70	1.46 (1.04–2.05)	0.0288	1.67 (1.19–2.36)	0.0030	2.71 (1.72–4.37)	< 0.0001	3.67 (2.30–6.00)	< 0.0001
Sex	Male/female	1.91 (1.36–2.72)	0.0002			2.42 (1.54–3.90)	0.0001	2.33 (1.47–3.78)	0.0003
Smoking status	Smoker/never smoked	1.52 (1.08–2.15)	0.0159			1.78 (1.15–2.80)	0.0097		
Grade	≥G2/G1	4.03 (2.73–6.13)	< 0.0001	2.31 (1.51–3.63)	< 0.0001	3.80 (2.30–6.60)	< 0.0001		
Stage	≥II/I	5.28 (3.75–7.47)	< 0.0001	3.30 (2.27–4.81)	< 0.0001	4.23 (2.73–6.59)	< 0.0001	2.78 (1.69–4.61)	< 0.0001
pl	Yes/no	3.47 (2.44–4.90)	< 0.0001			3.65 (2.34–5.65)	< 0.0001		
ly	Yes/no	4.95 (3.42–7.06)	< 0.0001	2.60 (1.75–3.82)	< 0.0001	4.28 (2.68–6.70)	< 0.0001	2.76 (1.65–4.53)	0.0001
v	Yes/no	3.08 (2.19–4.33)	< 0.0001			3.58 (2.31–5.56)	< 0.0001	1.83 (1.11–3.03)	0.0183
Histological subtype	Micropapillary or solid/others	2.06 (1.15–3.40)	0.0166			1.14 (0.44–2.42)	0.7572		
Surgical procedure	≥Lobectomy/sublobar resection	1.60 (1.04–2.56)	0.0322			1.66 (0.95–3.14)	0.0787		
EGFR*	Wild-type/mutant	1.76 (1.10–2.89)	0.0185			2.21 (1.18–4.40)	0.0128		
IL-38	High/low	1.75 (1.23–2.52)	0.0018			2.00 (1.26–3.26)	0.0030		

*cases for which data were available.

*P*-values for Cox proportional hazards regression analysis.

DFS: disease-free survival, OS: overall survival, pl: pleural invasion, ly: lymphatic invasion, v: vascular invasion, *EGFR*: epidermal growth factor receptor gene, IL-38: interleukin-38, HR: hazard ratio, CI: confidence interval.

### Association between PD-L1 and IL-38 expression in patients with primary lung adenocarcinoma

**Fig 3:**
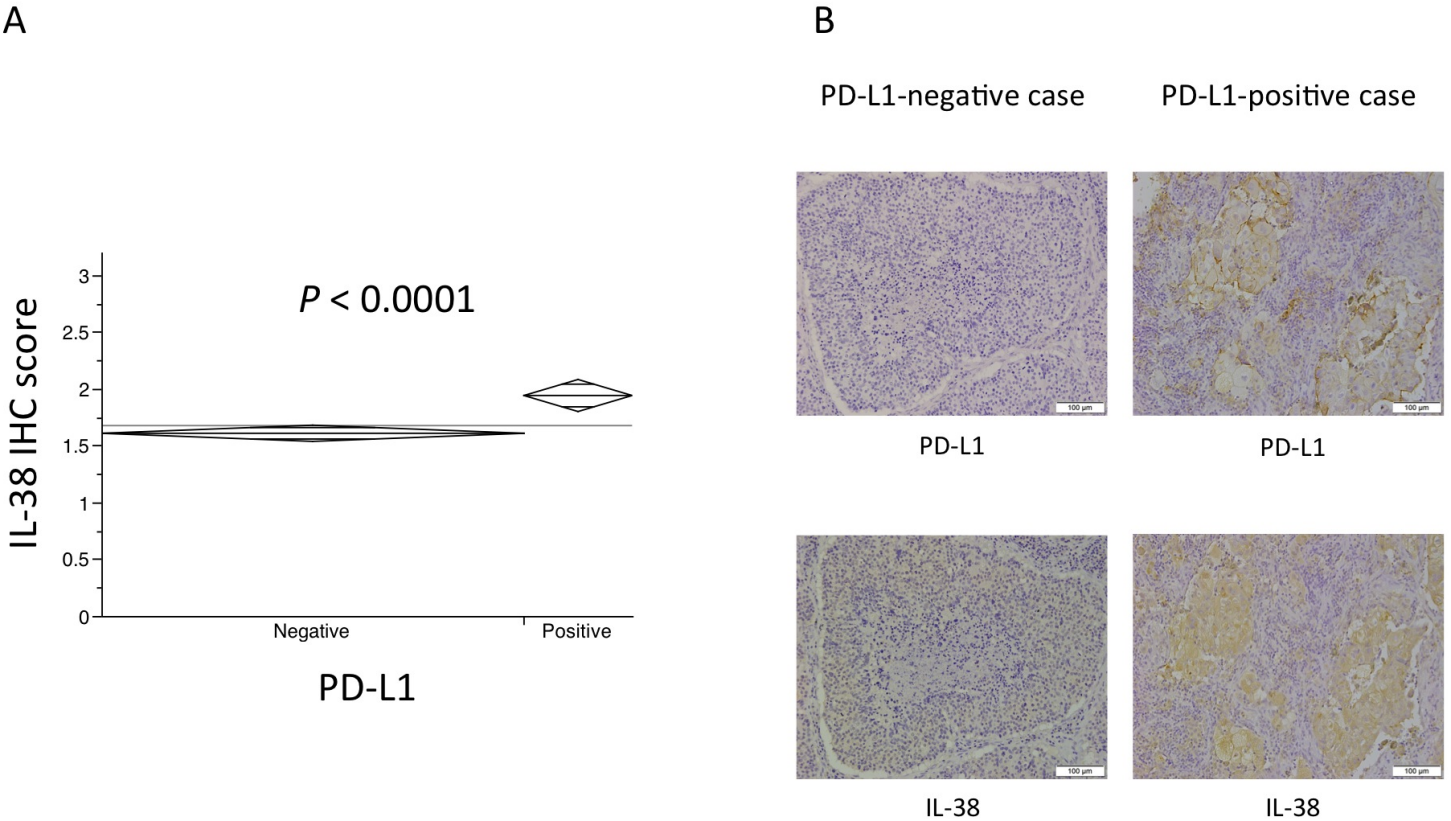
Association between PD-L1 and IL-38 expression. (A) PD-L1-positive cases showed higher expression of IL-38 than PD-L1-negative cases (Wilcoxon rank-sum test: *P* < 0.0001). (B) Representative images of (upper panel) PD-L1 staining and (lower panel) IL-38 staining in a (left panel) PD-L1-negative case and (right panel) PD-L1-positive case. IHC: immunohistochemistry, PD-L1: programmed cell death-ligand 1, IL-38: interleukin-38. Scale bar: 100 μm.

Next, we investigated the association between PD-L1 and IL-38 expression. PD-L1-positive cases showed higher expression of IL-38 than PD-L1-negative cases (Wilcoxon rank-sum test: *P* < 0.0001, **Fig 3A**). **Fig 3B** shows representative images of PD-L1 and IL-38 staining in a PD-L1-negative case and PD-L1-positive case.

### Disease-free and overall survival according to IL-38 expression in PD-L1-negative and PD-L1-positive cases

**Fig 4:**
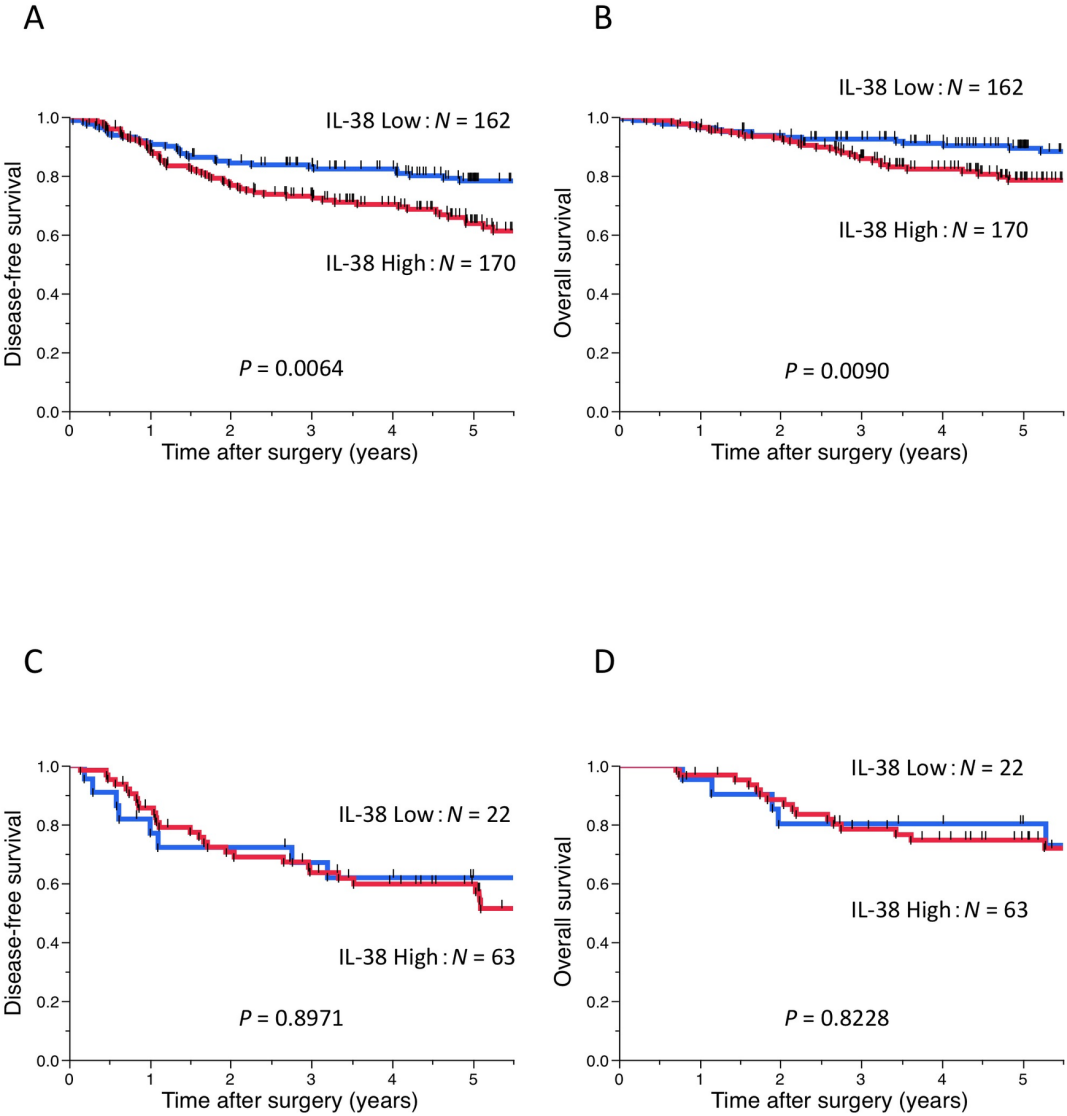
Kaplan-Meier curves according to IL-38 expression in the analysis of PD-L1-negative cases and PD-L1-positive cases. Kaplan-Meier curves showing (A, C) disease-free and (B, D) overall survival of primary lung adenocarcinoma patients according to IL-38 expression in the analysis of (A, B) PD-L1-negative cases and (C, D) PD-L1-positive cases. IL-38: interleukin-38, PD-L1: programmed cell death-ligand 1.

Finally, we assessed the associations between IL-38 expression and postoperative survival of patients in PD-L1-negative and PD-L1-positive cases, respectively. Survival analyses by the Kaplan-Meier method showed that patients with high expression of IL-38 had significantly shorter DFS and shorter OS after surgery than patients with low expression of IL-38 in the analysis of PD-L1-negative cases (log-rank test: *P* = 0.0064 and *P* = 0.0090, respectively, **Fig 4A and 4B**). However, in the analysis of PD-L1-positive cases, there was no significant association between IL-38 expression and postoperative survival of patients (log-rank test: DFS, *P* = 0.8971; OS, *P* = 0.8228, **Fig 4C and 4D**)

## Discussion

In present study, we identified increases in IL-38 expression of tumor cells in multiple cancer types and revealed that high expression was associated with poor prognoses of lung adenocarcinoma patients. This is the first report concerning the relevance between IL-38 expression and the prognosis of malignant tumors.

IL-6 is another cytokine expressed in tumor cells, and its dysregulation has been implicated in many diseases including malignant tumors for which it has been associated with tumor progression, drug resistance, and poor prognoses [30–36]. Huang et al. reported that the Janus kinase (Jak) 2/Signal transducer and activator of transcription (Stat) 3 pathway regulates autocrine production of IL-6 in lung cancer cells, and an IL-6 feed-forward loop plays an important role in the pathogenesis of inflammation-induced cancer [30]. However, in terms of IL-38, a mechanistic link between IL-38 expression and carcinogenesis, cancer growth, and poor prognoses remains unclear. IL-38 binds to the IL-36 receptor and inhibits its effect [11–14]. IL-36, which is constitutively and inducibly expressed on macrophages, dendritic cells, lymphocytes, skin, bronchial epithelium, and synovial fibroblasts, binds to the receptor constitutively expressed on dendritic cells and naive CD4^+^ T cells, resulting in cell proliferation, survival of naive T cells, and T helper (Th) 1 polarization [14, 37]. Therefore, Th1 differentiation may be downregulated in cases with high expression of IL-38, resulting in poor survival. Weinstein et al. proposed that IL-36 is involved in the formation of tertiary lymphoid organs in the tumor microenvironment [38]. The presence of tertiary lymphoid organs has been shown to be a biomarker for a good prognosis for cancer patients [38]. Moreover, some other previous reports have shown that IL-36 may have an anti-tumor activity and active CD8^+^ T cell responses [39, 40]. Therefore, IL-38 overexpression, which inhibits the effect of IL-36, may affect the tumor microenvironment and lead to a poor prognosis. We should examine the relationship between IL-38 expression and the infiltration of immune-related cells such as CD3^+^, CD4^+^, and CD8^+^ cells to confirm the above points in future studies. Additionally, Lv et al. have recently reported that tumor-associated transcriptional factors c-Fos, activator protein-1, c-Jun, and nuclear factor κB bind to the upstream region of the IL-36RN gene that encodes the anti-inflammatory cytokine IL-36Ra [41]. This finding may indicate that IL-38 is involved in carcinogenesis and tumor progression through the regulation of tumor-associated transcriptional factors.

We investigated the relationship between PD-L1 and IL-38 expression. PD-L1-positive cases showed higher expression of IL-38 than PD-L1-negative cases. PD-1 is a receptor expressed on the surface of activated T cells, mainly CD8^+^ T cells, and PD-1/PD-L1 interactions act as an immune checkpoint signal, suppressing the effector functions of activated T cells [1, 42, 43]. PD-L1-expressing cases may also express IL-38 and suppress not only CD8^+^ T cells, but also CD4^+^ T cells. In the present study, multivariate analysis did not show that high expression of IL-38 remained as a predictor for DFS and OS (**Table 4**). Furthermore, survival analyses by the Kaplan-Meier method showed that patients with high expression of IL-38 had significantly shorter DFS and shorter OS after surgery than patients with low expression of IL-38 in the analysis of PD-L1-negative cases. However, in the analysis of PD-L1-positive cases, there was no significant association between IL-38 expression and postoperative survival of patients (**Fig 4**). Based on these findings, the prognostic significance of IL-38 may be weaker than that of PD-L1. However, IL-38 expression may be a new biomarker for PD-1/PD-L1 inhibitors.

The present study had several limitations. First, this was a single institutional retrospective study and not a trial-based correlative study. However, this is the first report concerning the relevance between IL-38 expression and the prognosis of malignant tumors. The second limitation was that we examined IL-38 expression only in lung adenocarcinomas. Further study of the association between IL-38 expression and clinicopathological characteristics and prognoses of other histological types of lung cancers and other cancer types is therefore required.

In conclusion, IL-38 was expressed in tumor cells of various cancers including primary lung adenocarcinoma. Moreover, IL-38 expression was significantly associated with poor survival of lung adenocarcinoma patients. IL-38 may affect host immunity or the tumor microenvironment, and contribute to the progression of lung adenocarcinoma.

## Supporting information

S1 Fig Representative images of (left panel) HE staining, (middle panel) IL-38 staining, and (right panel) negative controls. (A) Lung adenocarcinoma. (B) Lung squamous cell carcinoma. (C) Lung small cell carcinoma. (D) Esophageal cancer. (E) Gastric cancer. (F) Colon cancer. (G) Hepatocellular carcinoma. (H) Breast cancer. These results indicated expression of IL-38 in tumor cells of multiple cancer types. HE: Hematoxylin-eosin, IL-38: interleukin-38. Scale bar: 100 μm.
(TIF)
