# Supplementary figures and images for: Clinical implications of the novel cytokine IL-38 expressed in lung adenocarcinoma: Possible association with PD-L1 expression
Source: PLoS One. 2017 Jul 20;12(7):e0181598. doi: 10.1371/journal.pone.0181598 (PMC5519175; doi:10.1371/journal.pone.0181598)

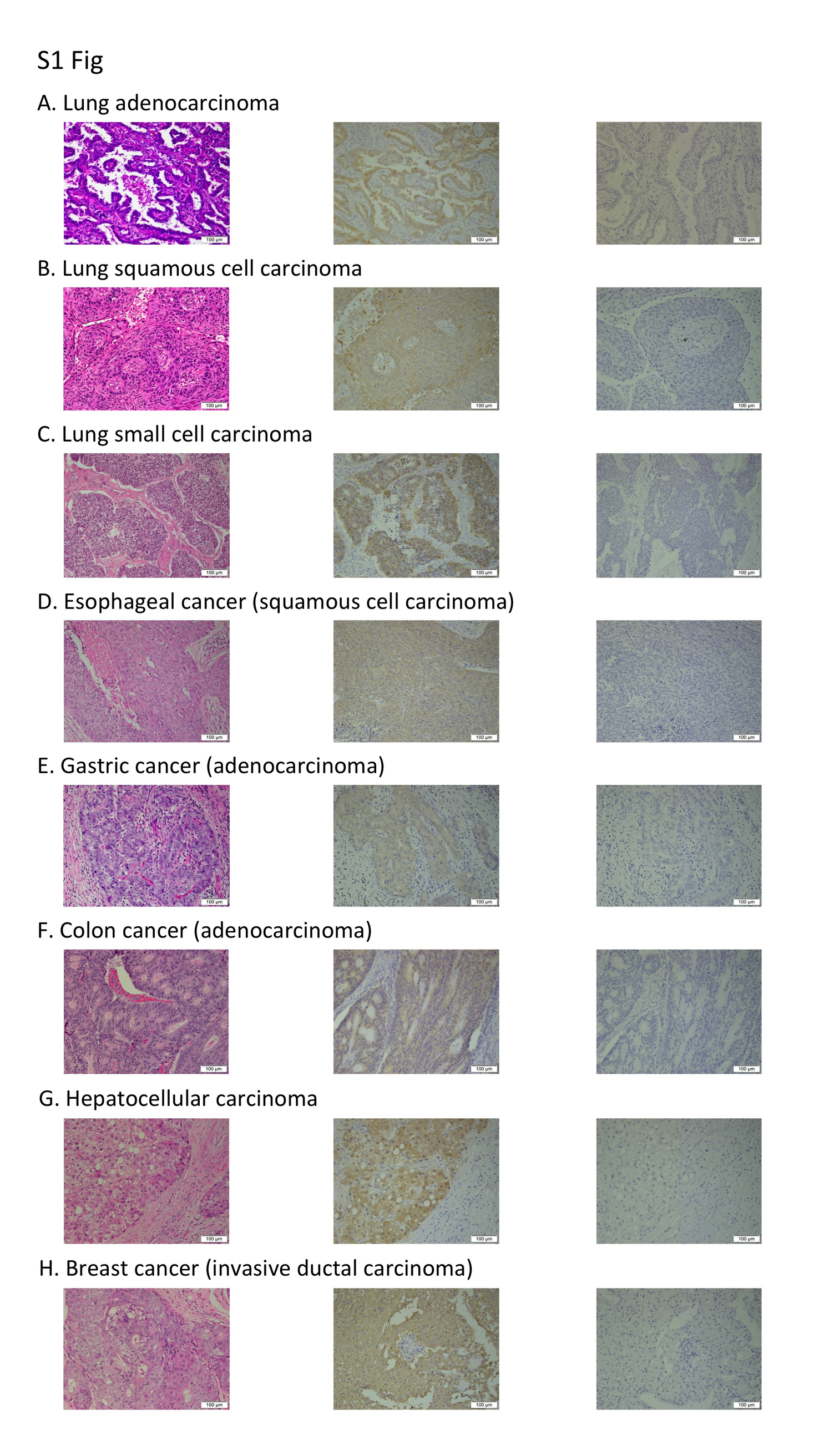

Supplement: S1 Fig — (A) Lung adenocarcinoma. (B) Lung squamous cell carcinoma. (C) Lung small cell carcinoma. (D) Esophageal cancer. (E) Gastric cancer. (F) Colon cancer. (G) Hepatocellular carcinoma. (H) Breast cancer. These results indicated expression of IL-38 in tumor cells of multiple cancer types. HE: Hematoxylin-eosin, IL-38: interleukin-38. Scale bar: 100 μm. (TIF) [file pone.0181598.s001.tif]
